# Subclinical myocardial changes in idiopathic premature ventricular contractions: insights from cardiac magnetic resonance imaging

**DOI:** 10.3389/fcvm.2026.1650580

**Published:** 2026-02-19

**Authors:** Yoshinao Yazaki, Hisanori Kosuge, Masatake Kobayashi, Tomoya Okano, Yasuyuki Takada, Takahiro Kusume, Muryo Terasawa, Natsuko Inagaki, Shuichiro Kazawa, Kazuhiro Satomi

**Affiliations:** 1 Department of Cardiology, Tokyo Medical University Hospital, Tokyo, Japan; 2 Department of Clinical Genetics Center, Tokyo Medical University Hospital, Tokyo, Japan

**Keywords:** cardiac magnetic resonance imaging, late gadolinium enhancement, premature ventricular contractions, structural heart disease, T1 value

## Abstract

**Background:**

Idiopathic premature ventricular contractions (PVCs) are defined as PVCs in the absence of obvious structural heart disease, which is typically excluded by electrocardiography (ECG), echocardiography. The purpose of this study was to investigate the prevalence of subclinical myocardial changes detected by cardiac magnetic resonance imaging (CMRI) in patients with idiopathic PVCs.

**Methods:**

In this cross-sectional study, thirty-three patients (age 54 ± 17 years, 19 male) with idiopathic PVCs diagnosed using ECG and echocardiography who subsequently underwent CMRI at 3-Tesra between September 2019 and May 2024 were included in this study. CMRI, including late gadolinium enhancement (LGE), native T1 values, and extracellular volume fraction (ECV), was performed.

**Results:**

PVCs most frequently originated from the right ventricular outflow tract (15 of 33 patients, 45%). CMR identified myocardial changes in 17/33 (52%). LGE was present in 11/33 (33%). Importantly, mapping-derived indices (native T1 values/ECV) revealed subclinical interstitial changes in several patients, including those without LGE (four with elevated native T1 and two with elevated ECV). Among patients with myocardial changes, the incidence of non-sustained ventricular tachycardia and the maximum consecutive number of PVCs were significantly higher than in those without myocardial changes (*P* < 0.05 for both).

**Conclusions:**

In this exploratory study, multiparametric CMRI frequently revealed subclinical myocardial changes in patients with idiopathic PVCs despite normal ECG and echocardiography. Given the small sample size and the heterogeneous, ECG-based estimation of PVC origin, these findings should be considered hypothesis-generating, and larger studies are warranted.

## Graphical Abstract

**Figure:**
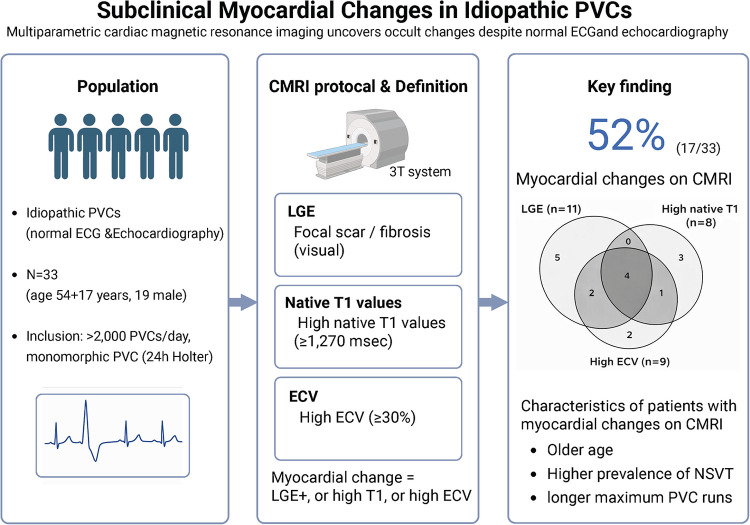

## Introduction

Premature ventricular contractions (PVCs) are among the most frequent arrhythmias identified in daily clinical practice. In these patients, further investigations, such as electrocardiography (ECG), echocardiography are required to exclude structural heart diseases (1). When structural abnormalities are excluded, patients are diagnosed with idiopathic PVCs and usually have a favorable prognosis (2). However, rare cases of sudden cardiac death have been reported in patients labeled as having idiopathic PVCs, where postmortem examination later revealed underlying cardiomyopathy (3). Earlier identification of such malignant cases should be required but sometimes challenging. Muser et al. found late gadolinium enhancement (LGE) on CMRI, which represents tissue fibrosis, in 18% of idiopathic PVC patients (4). These PVCs patients with LGE had a worse prognosis than patients without LGE. In addition to LGE, native T1 values and extracellular volume fraction (ECV) on CMRI have recently been considered as parameters with high sensitivity to detect subclinical myocardial changes, even in early-stages cardiomyopathies (5, 6). The study aimed to evaluate the utility of CMRI including LGE, native T1 values, and ECV to detect early-phase structural heart disease in patients with idiopathic PVCs in whom no abnormalities were detected on ECG, echocardiography. We hypothesized that multiparametric CMRI (LGE, native T1 values, and ECV) would detect subclinical myocardial changes and that these changes would be associated with arrhythmic complexity.

## Methods

### Study patients

This was a single-center cross-sectional observational study conducted at Tokyo Medical University Hospital (Tokyo, Japan). Thirty-three patients with idiopathic PVCs who subsequently underwent CMRI at 3-tesra between September 2019 and May 2024 were included in this study. Idiopathic PVCs were defined as PVCs in which structural heart disease had been excluded by standardized diagnostic assessment consisting of physical examinations, laboratory analysis, chest x-rays, ECG, and echocardiography other than CMRI. Patients with daily PVC counts exceeding 2,000 with a monomorphic QRS waveform on 24-h Holter ECG were included in the present analysis. Patients who had more than moderate valvular disease, low left ventricular ejection fraction (LVEF) (less than 50%), and dilated ventricular chambers (more than 55 mm of ventricular internal dimension in diastole) on echocardiography were excluded. Patients with any ECG findings suggestive of structural heart disease or a known history of heart disease were also excluded. All patients included in this registry provided written, informed consent to participate in the registry. The study was approved by the Institutional Review Board of Tokyo Medical University (approval number, T2023-0138).

### Cardiac magnetic resonance imaging protocol and analysis

**Figure 1:**
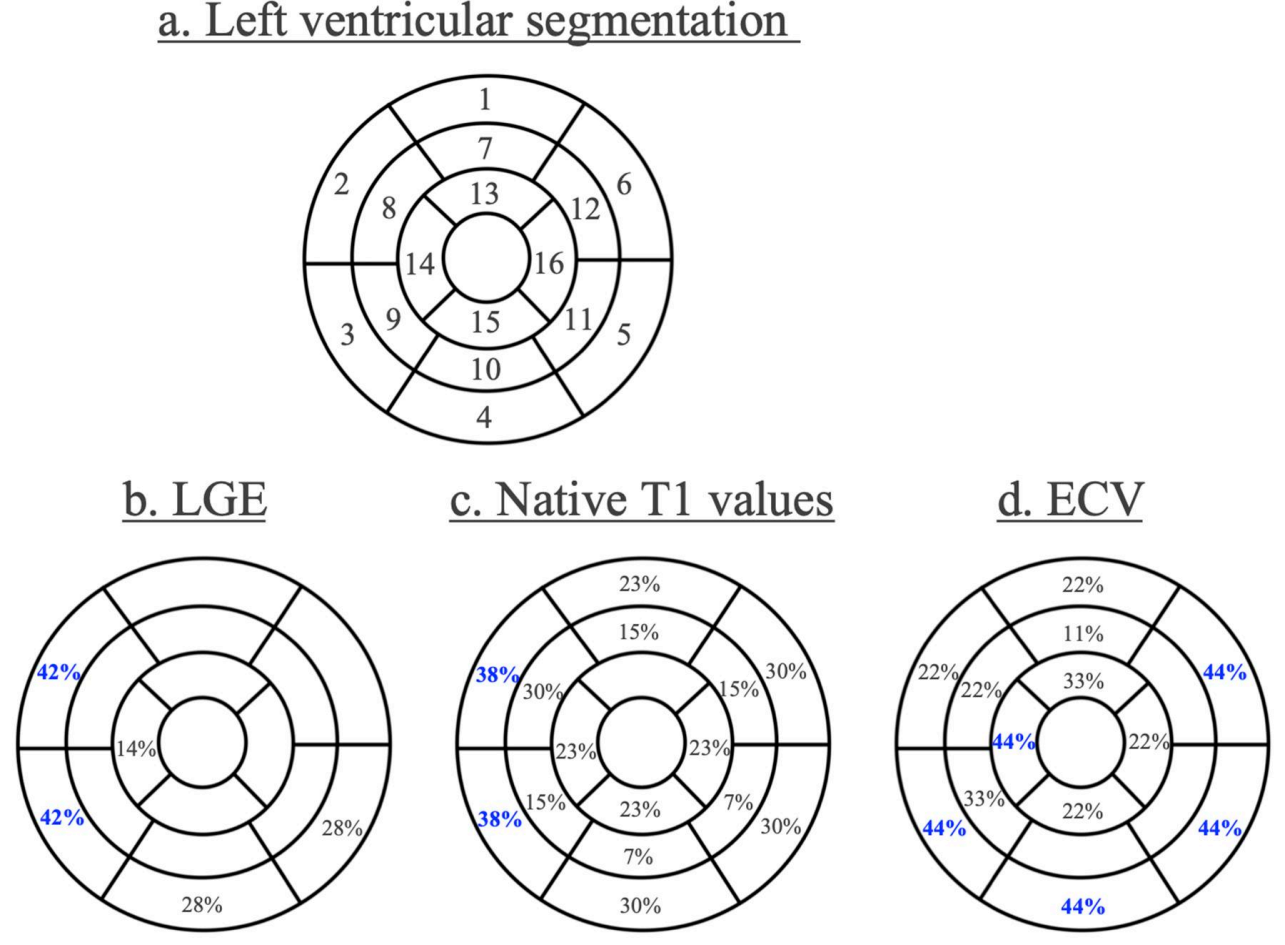
Left ventricular segmentation and distribution of myocardial changes. **(a)** Display, on a circumferential polar plot, of 17 myocardial segments. (1) Basal anterior (2) basal anteroseptal (3) basal inferoseptal (4) basal inferior (5) basal inferolateral (6) basal anterolateral (7) mid anterior (8) mid anteroseptal (9) mid inferoseptal (10) mid inferior (11) mid inferolateral (12) mid anterolateral (13) apical anterior (14) apical septal (15) apical inferior (16) apical lateral. **(b–d)** Distribution of LGE **(b)**, native T1 values **(c)**, and ECV **(d)** on CMRI in patients with idiopathic PVCs. Blue indicates the most frequent location of myocardial changes. CMRI, cardiac magnetic resonance imaging; LGE, late gadolinium enhancement; ECV, extracellular volume fraction; PVCs, premature ventricular contractions.

All patients underwent CMRI using a Magnetom Skyra 3 T system (Siemens Healthineers, Erlangen, Germany) with a 60-channel body coil. Cine images of three long-axis slices (2-chamber, 3-chamber, and 4-chamber) and short-axis slices covering the whole left ventricle were acquired using steady-state free precession [repetition time [TR]: 28.2 ms, echo time [TE]: 1.6 ms, flip angle: 60°, field-of-view [FOV]: 360 × 270 mm, acquisition matrix: 224 × 224, slice thickness: 6 mm]. Native T1 mapping was performed on 3 short-axis slices (base, mid-ventricle, and apex) using a modified Look-Locker Inversion recovery (MOLLI) sequence with a 5(3)3 scheme (TR: 349 ms, TE: 1.1 ms, flip angle: 35°, FOV: 360 × 306 mm^2^, acquisition matrix: 256 × 169, slice thickness: 8 mm). Post-contrast T1 mapping was performed 10–15 min after intravenous injection of 0.1 mmol/kg gadobutrol (Gadovist, Bayer Healthcare, Leverkusen, Germany) using the same protocol as for native T1 mapping. Then, LGE images were acquired using an inversion-recovery gradient echo sequence (TR: 700 ms, TE: 1.9 ms, flip angle: 12°, FOV: 360 × 270 mm^2^, acquisition matrix: 192 × 157, slice thickness: 6 mm). The arrhythmia rejection functionality was used to improve image quality. The CMRI were analyzed by two independent experienced observers using commercially available computer software (Ziostation 2, Ziosoft, Tokyo, Japan). LVEF and LV volume and mass were manually measured using short-axis cine images (7). The left ventricle was divided into 16 segments on the short-axis view, and each location was named as shown in Figure 1a (8). Native T1 and post-contrast T1 values were determined by drawing a region of interest in each segment. ECV in each segment was calculated as follows: ECV = (1-hematocrit) × (1/post-contrast T1 of the myocardium—1/native T1 of the myocardium)/(1/post-contrast of the blood—1/native T1 of the blood). Hematocrit was measured on the same day as CMRI. Myocardial changes on CMRI were defined as the presence of one of LGE, ≥3 segments with high native T1 values (≥1,270 ms), or ≥3 segments with high ECV (≥30%). The presence of LGE was determined by visual examination. Furthermore, we distinguished overt myocardial fibrosis (LGE) from subclinical myocardial changes (high native T1 value/high ECV), the latter reflecting diffuse interstitial remodeling. Several factors including age, sex, ECG parameters, 24-h Holter ECG data, and echocardiography findings were compared between PVCs patients with and without myocardial changes on CMRI to identify the features of patients with myocardial changes.

### Statistical analysis

Categorical variables are presented as frequencies (percentages), and continuous variables are presented as means ± standard deviation or medians (25th and 75th percentiles), depending on the distribution. Comparisons of the characteristics of patients with and without myocardial changes on CMRI were made using the Mann–Whitney *U* and *χ*^2^ tests, as appropriate.

Linear regression analysis was performed to assess the associations between myocardial changes on CMRI and maximum PVC run counts. The model was adjusted for the following covariates: age, symptoms when PVCs were documented (i.e., palpitations and dizziness), B-type natriuretic peptide, and the presence of left bundle branch block (LBBB)-type PVCs.

Statistical analyses were performed using R version 3.4.0 (R Development Core Team, Vienna, Austria). A two-sided *p*-value <0.05 was considered significant. Owing to the limited sample size, no prespecified subgroup, interaction, or sensitivity analyses were performed. There were no missing data for variables reported in Tables 1–4.

**Table 1:** Patients’ characteristics.

Characteristics	Overall (*N* = 33)
Age, years	54 ± 17
Male, *N* (%)	19 (57.6%)
Symptoms, *N* (%)	13 (39.4%)
Hypertension, *N* (%)	11 (33.3%)
Medications, *N* (%)	
Beta-blockers	4 (12.1%)
ACEis or ARBs	5 (15.2%)
CCBs	9 (27.3%)
Antiarrhythmic drugs	0 (0%)
BNP, pg/mL	30 (15–46)
Origin of PVCs	
LVi	5 (15.2%)
RVi	3 (9.0%)
LVOT	8 (24.2%)
RVOT	15 (45.5%)
Septal	2 (6.0%)

Values are expressed as means ± SD, *n* (%) or medians (25th to 75th percentile).

ACEi, angiotensin converting enzyme inhibitor; ARB, angiotensin receptor blocker; CCB, calcium channel blocker; BNP, B-type natriuretic peptide; PVCs, premature ventricular contractions; LVi, left ventricular inferior; RVi, right ventricular inferior; LVOT, left ventricular outflow tract; RVOT, right ventricular outflow tract.

**Table 2:** Cardiac magnetic resonance imaging.

Cardiac MRI	
LGE, *N* (%)	11 (33.3%)
Native T1, ms	1,199.0 ± 35.7
ECV (%)	25.0 ± 3.4
LVEF, %	53.2 ± 5.7
LVDVi, mL/m^2^	70.1 ± 14.5
LVMi, g/m^2^	37.8 ± 9.3

Values are expressed as means ± SD, *n* (%) or medians (25th to 75th percentile).

LGE, late gadolinium enhancement; ECV, extracellular volume fraction; LVEF, left ventricular ejection fraction; LVDVi, left ventricular end-diastolic volume index; LVMi, left ventricular mass index.

**Table 3:** Myocardial changes on cardiac magnetic resonance imaging and patients’ characteristics and data.

Characteristics	Myocardial changes on CMRI (*N* = 17)	Normal on CMRI (*N* = 16)	*P*-value
Age, years	62 ± 13	45 ± 15	**0** **.** **003**
Male, *N* (%)	10 (55.6%)	9 (60.0%)	0.797
Symptoms, *N* (%)	5 (27.8%)	8 (53.3%)	0.135
Hypertension, *N* (%)	8 (44.4%)	3 (20.0%)	0.138
BNP, pg/mL	33 (16–52)	29 (6–46)	0.485
PVC LBBB type, *N* (%)	10 (55.6%)	9 (60.0%)	0.797
PVC inferior axis, *N* (%)	14 (77.8%)	11 (73.3%)	1
Total counts of PVCs, beats/day	13,918 (8,439–20,660)	21,353 (11,241–24,406)	0.117
PVC burden, %	14.1 (8.0–19.0)	18.0 (11.0–27.0)	0.187
PVC burden ≥20%, *N* (%)	4 (22.2%)	6 (40.0%)	0.468
NSVT, *N* (%)	15 (83.3%)	5 (33.3%)	**0**.**003**
Maximum consecutive number of PVCs	5.7 ± 5.6	2.4 ± 0.6	**0**.**0008**
Echocardiogram			
LVEF, %	64.7 ± 3.9	60.5 ± 5.4	0.07
LVDD, mm	46.4 ± 4.3	47.6 ± 4.0	0.37
TAPSE, mm	25.5 ± 3.8	25.4 ± 4.4	0.94
RVSP, mmHg	21.0 ± 6.1	19.6 ± 5.4	0.56
CMRI			
LGE, *N* (%)	11 (61.1%)	0 (0.0%)	**0**.**0002**
Native T1, ms	1,214.8 ± 35.8	1,184.6 ± 30.2	0.084
ECV (%)	26.2 ± 4.0	23.7 ± 2.2	**0**.**044**
LVEF, %	52.8 ± 4.0	53.8 ± 7.4	0.823
LVDVi, mL/m^2^	67.8 ± 13.8	72.6 ± 15.3	0.414
LVMi, g/m^2^	39.8 ± 10.4	35.6 ± 7.7	0.355

Values are expressed as means ± SD, *n* (%) or medians (25th to 75th percentile).

Bold values indicate statistical significance (*P* < 0.05).

BNP, B-type natriuretic peptide; PVCs, premature ventricular contractions; CLBBB, complete left bundle branch block; NSVT, non-sustained ventricular tachycardia; ECV, extracellular volume fraction; LVEF, left ventricular ejection fraction; LVDD, left ventricular end-diastolic diameter; TAPSE, tricuspid annular plane systolic excursion; RVSP, right ventricular systolic pressure; CMRI, cardiac magnetic resonance imaging; LGE, late gadolinium enhancement; LVDVi, left ventricular end-diastolic volume index; LVMi, left ventricular mass index.

**Table 4:** Association between myocardial changes on cardiac magnetic resonance imaging and maximum consecutive number of PVCs.

Variable	Univariable		Multivariable	
Myocardial changes on CMRI	beta (95% CI)	*p*-value	beta (95% CI)	*p*-value
	3.31 (0.35–6.26)	0.03	4.47 (0.18–8.76)	0.042

CMRI, cardiac magnetic resonance imaging; PVCs, premature ventricular contractions**.**

## Results

### Patients' characteristics

Thirty-three patients (age 54 ± 17 years, 19 male) with idiopathic PVCs diagnosed using ECG and echocardiography were included in this study. Physical examinations, laboratory tests, and chest x-rays showed no significant abnormalities in all patients. The characteristics of the population are summarized in Table 1. The majority of patients were referred to our hospital after frequent PVCs were incidentally detected during routine medical check-ups. Symptoms, such as palpitations, shortness of breath, and chest discomfort, were present in 39% of all patients. One patient experienced presyncope and was diagnosed with neurally mediated syncope. None of the patients had a previously diagnosed structural heart disease or a family history of sudden cardiac death.

### Electrocardiography and echocardiography at baseline

All patients underwent a 12-lead ECG and echocardiography. All patients had no abnormality on ECG suggestive of structural heart disease. All patients were in sinus rhythm at baseline. Three patients had nonspecific ST-T changes on ECG; in each of these cases, subsequent echocardiography and computed tomography coronary angiography (CTCA) were normal. Two patients had complete right bundle branch block on the baseline ECG. No patients had any degree of atrio-ventricular block or a prolonged QT interval.

All patients underwent echocardiography prior to undergoing CMRI. In all patients, left ventricular wall motion was normal, LVEF was preserved (62.8 ± 5.0%), and there was no left ventricular dilatation (LV end-diastolic diameter 47.0 ± 4.1 mm). No significant valvular disease was present, and none of the patients had evidence of pulmonary hypertension on echocardiography.

### 24-h Holter electrocardiography

All patients underwent 24-h Holter ECG. The median number of PVCs was 17,450 (10,338–23,376) beats per day. A median daily PVCs burden was 17.5% (10.0%–20.1%). Non-sustained ventricular tachycardia (NSVT), defined as more than 3 consecutive beats of PVCs and lasting less than 30 s, was documented in 20 (61%) patients. The median number of maximum consecutive PVCs in each patient was 3 beats. All patients had documented monomorphic PVCs. Based on ECG-derived categories, PVC origins were RVOT 45.5%, LVOT 24.2%, left-ventricular inferior 5.2%, right-ventricular inferior 9.0%, and septal 6.0%. We did not further subdivide septal sites into para-Hisian vs. outflow-tract septum or distinguish papillary muscle origins within the LV inferior group because invasive mapping was not available in most cases.

### Cardiac magnetic resonance imaging

**Figure 2:**
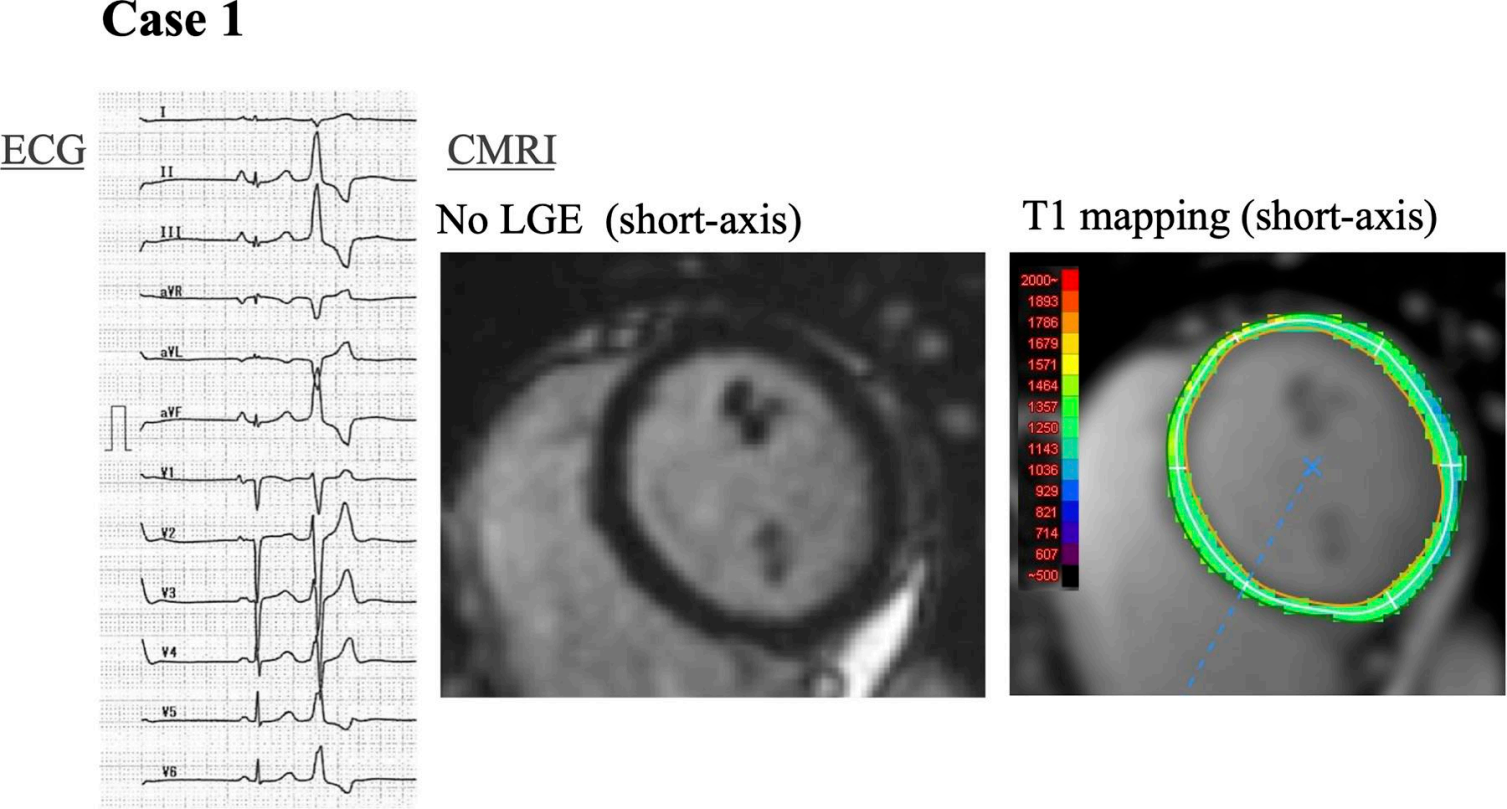
A 40-year-old woman with frequent PVCs. Case 1: a 40-year-old woman with frequent monomorphic PVCs that originated from the right ventricular outflow tract. No LGE on CMRI, but diffuse high native T1 values (maximum 1,343 ms) in the left ventricle. PVCs, premature ventricular contractions; LGE, late gadolinium enhancement; CMRI, cardiac magnetic resonance imaging.

**Figure 3:**
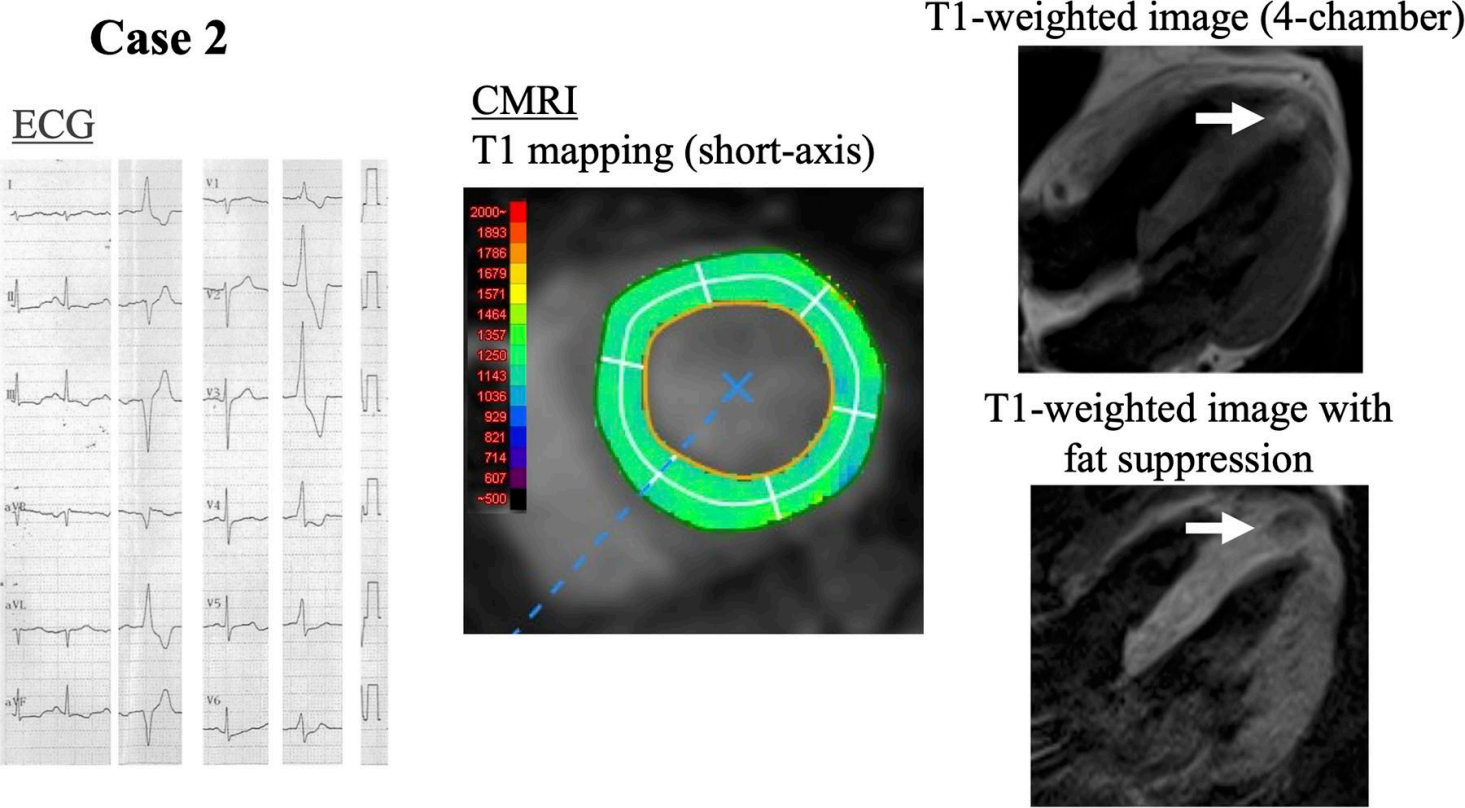
A 40-year-old woman with suspected ALVC. Case 2: a 40-year-old woman with monomorphic PVCs that originated from the left ventricular inferior wall. No LGE on CMRI, but high native T1 values (maximum 1,302 ms). T1-weighted image with fat suppression shows intramyocardial fat signal of suspected ALVC (white arrow). PVCs, premature ventricular contractions; LGE, late gadolinium enhancement; CMRI, cardiac magnetic resonance imaging; ALVC, arrhythmogenic left ventricular cardiomyopathy.

In 17 of 33 patients (52%), myocardial changes were detected on CMRI including LGE, high native T1 values, or high ECV. LGE was seen in 11 (33%) patients, a high native T1 value was seen in 8 (24%) patients, and high ECV was present in 9 (27%) patients. The segmental distribution of these myocardial changes is shown in Figures 1b–d. The most frequent location of LGE was basal anteroseptal and inferoseptal in 42% of patients (Figure 1b). No right ventricular LGE was observed in any patient. A high native T1 value was most often localized in the basal septal region in 38% of patients (Figure 1c), and a high ECV was located in a basal location and apical septal region (44%) (Figure 1d). Notably, 4 patients showed no evidence of LGE on CMRI but had diffusely elevated native T1 values. Two representative cases are presented in which LGE was absent while T1 mapping identified myocardial changes. Figures 2, 3 shows a patient with idiopathic PVCs in whom CMRI demonstrated myocardial changes despite negative LGE. One patient was a 40-year-old woman who was referred to our hospital because of frequent PVCs on ECG at an annual health check-up. She was asymptomatic, and baseline ECG and echocardiography showed no abnormalities. The patient underwent CMRI for further investigation. There was no LGE enhancement, but native T1 values were high (Figure 2). Another PVCs case shown in Figure 3 was a 40-year-old woman with normal ECG and echocardiography at baseline. There was no LGE, but native T1 values were high, as in the previous case. T1-weighted images showed an intramyocardial fat signal in the left ventricle and suggested arrhythmogenic left ventricular cardiomyopathy (ALVC).

In this study, all patients had preserved function of both ventricles, no chamber dilatation, and no abnormal wall motion on CMRI. The detailed CMRI data are shown in Table 2. Of the 17 patients who had myocardial changes on CMRI, 11 patients underwent CTCA, and no coronary artery stenosis was observed in these patients.

### Correlation between cardiac magnetic resonance imaging and patients' characteristics

Patients with myocardial changes on CMRI were significantly older than those without (62 ± 13 years vs. 45 ± 15 years; *P* < 0.05). There were no significant differences between the two groups in terms of PVC site of origin, total number of PVCs, PVC burden, or echocardiographic parameters such as LVEF and chamber sizes (Table 3). Proportion of patients who had NSVT was significantly higher in the CMRI-myocardial changes group than in the CMRI-normal group (83% vs. 33%; *P* < 0.05). Additionally, the maximum consecutive number of PVCs was higher in patients with myocardial changes compared to those without (mean 5.7 vs. 2.4 beats; *P* < 0.05). This difference in PVC run length remained statistically significant in a multivariable linear regression analysis adjusting for age, symptoms, BNP level, and PVC morphology (Table 4). No other clinical or ECG characteristics were significantly associated with the presence of myocardial changes on CMRI.

## Discussion

In this study of patients with idiopathic PVCs (i.e., PVCs without apparent structural heart disease on standard evaluation), we found that: (1) CMRI, utilizing LGE, native T1 mapping, and ECV, identified myocardial changes in approximately half (52%) of the patients; (2) patients with positive CMRI findings were older and had a higher prevalence of NSVT as well as longer runs of PVCs compared to those with normal CMRI.

The definition of idiopathic PVCs implies the exclusion of underlying structural heart disease by routine diagnostic tools such as ECG, echocardiography. Idiopathic PVCs presumed to arise from the RVOT are often benign and have been associated with favorable long-term outcomes. Typically, PVCs with a left bundle branch block morphology and inferior axis (consistent with an RVOT origin) are considered benign idiopathic PVCs and have been associated with favorable long-term outcomes (2). However, isolated case reports and series have documented that some patients diagnosed with idiopathic PVCs can later be found to have structural disorders—such as arrhythmogenic right ventricular cardiomyopathy (ARVC)—when evaluated with more sensitive modalities like CMRI or cardiac CT (9). Our data indicate that even in a population stringently screened to exclude heart disease, by adding LGE in addition to native T1 value and ECV measurements, CMRI can uncover latent subclinical myocardial changes in about half of the cases. Thus, CMRI offers added value in the work-up of frequent PVCs, functioning as a sensitive tool to unmask subtle forms of cardiomyopathy that are not detected by conventional investigations.

### Utility of cardiac magnetic resonance imaging including LGE, native T1 values, and ECV

Cardiac MRI is uniquely sensitive for detecting myocardial fibrosis and other structural abnormalities, even in early or subclinical stages, compared to echocardiography or CTCA. LGE imaging, in particular, identifies focal myocardial scar or fibrosis and has become a reference standard for diagnosing both ischemic and non-ischemic cardiomyopathies (10, 11). In a prior CMRI study of patients with idiopathic PVCs, 16% of patients showed LGE (4). In our study, we extended the CMRI evaluation to include T1 mapping and ECV, which can detect diffuse myocardial changes beyond the spatial resolution of LGE. Although LGE has become the reference standard imaging for the diagnosis of cardiomyopathy, evaluating diffuse fibrosis or microscopic interstitial fibrosis could be limited because of the spatial resolution of LGE images. To overcome this limitation of LGE imaging, native T1 values and ECV have been proposed. T1 mapping calculates the longitudinal or spin-lattice relaxation time, which is determined by how rapidly protons re-equilibrate their spins after being excited by a radiofrequency pulse. The native T1 value lengthens with interstitial expansion caused by edema, infarction, amyloid infiltration, and fibrosis. ECV is also a marker of myocardial tissue remodeling, with a value of 25.3 ± 3% reported in healthy subjects, and it provides a physiologically intuitive unit of measurement (12). Both the native T1 value and ECV had higher sensitivity to detect abnormalities in patients with amyloidosis than LGE imaging (13, 14). In the present study, myocardial changes on CMRI were found in 52% of patients with idiopathic PVCs. The prevalence of myocardial changes was much higher than in a previous study because the native T1 values and ECV might have contributed to this result. Four patients had no LGE but high native T1 values, and two patients had no LGE but a high ECV. The native T1 value and ECV could be more useful to identify myocardial changes in the early stages of heart diseases than LGE. Further studies are needed to establish diagnostic cut-offs and to assess the specificity of native T1 and ECV.

**Figure 4:**
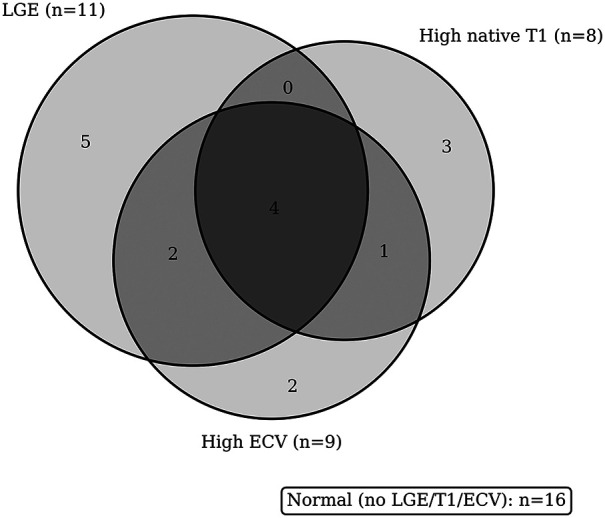
Overlap of myocardial changes on CMRI. Venn diagram showing the overlap among LGE, high native T1, and high ECV abnormalities. Counts indicate the number of patients in each region. Totals: LGE = 11, T1 = 8, ECV = 9; normal (no LGE/T1/ECV) = 16.

Consistent with our Results, several patients had mapping abnormalities in the absence of focal scar—specifically, four with elevated native T1 values and two with elevated ECV despite negative LGE—supporting the concept that T1/ECV mapping can detect diffuse interstitial remodeling beyond the spatial resolution of LGE. Conversely, 16 patients were normal across all three indices. Taken together, these patterns highlight the incremental yield and complementary pathophysiological information provided by a multiparametric CMR protocol in idiopathic PVCs (Figure 4).

### Characteristics of patients with myocardial changes on CMRI

We observed that patients with myocardial changes on CMRI were, on average, older than those without myocardial changes. Interestingly, a study of healthy volunteers found that ECV is relatively independent of age, while native T1 tends to decrease slightly with healthy aging (15). Our finding of older age in the CMRI-positive group might reflect that older patients had a longer duration of PVCs or a longer cumulative exposure to whatever process is causing the myocardial changes. Aside from age, the standard clinical and echocardiographic parameters did not differ between CMRI-positive and CMRI- negative patients, highlighting that the CMRI findings were truly occult—not evident from other examinations.

Holter monitoring data did reveal differences: patients with myocardial changes on CMRI were much more likely to have NSVT and higher maximum consecutive number of PVCs. Total PVC frequency or burden, however, was not significantly different between groups. This suggests that it is not just the quantity of PVCs, but the propensity for PVCs to become repetitive or sustained (as in NSVT) that correlates with underlying myocardial fibrosis on CMRI. It is plausible that the presence of myocardial fibrosis or scar provides an arrhythmogenic substrate that facilitates runs of PVCs or NSVT. Therefore, in idiopathic PVC patients, the occurrence of NSVT or long PVC runs might be a red flag prompting further investigation such as CMRI for underlying cardiomyopathy. Clinically, our results emphasize the importance of diligent follow-up in patients with idiopathic PVCs who exhibit such arrhythmic features. We would advocate periodic ECG/Holter monitoring in these patients to watch for progression of ventricular arrhythmias, and consideration of follow-up CMRI to monitor any evolution of myocardial abnormalities.

### Distribution of CMRI findings

Muser et al. found myocardial changes in the inferior-posterior basal wall most frequently in patients with idiopathic PVCs (4). However, in the present study, the most predominant area showing LGE was the septal basal wall (Figure 1b). A high native T1 value was most frequently observed in the basal wall, whereas a high ECV was seen in the basal inferior and lateral wall (Figures 1c,d). The present distribution of LGE was different from the previous study, but the basal wall was a common area in both studies. The reasons for this basal predilection are unclear, but one could speculate about differences in myocardial strain or perfusion in basal segments. The RVOT as a predominant origin of PVCs was not correlated with the area showing myocardial changes on CMRI in the present study. More broadly, the absence of an apparent anatomical concordance between the distribution of CMRI abnormalities and the presumed PVC origin may reflect several factors. First, CMRI abnormalities may represent an arrhythmogenic substrate, whereas the triggering focus of PVCs can arise from adjacent or remote myocardium and therefore may not coincide anatomically with the substrate. Second, LGE is optimized for detecting macroscopic focal scar and may miss microscopic/border-zone changes or diffuse interstitial remodeling, and subtle pathology in the RVOT is technically challenging to detect because of the thin right ventricular wall. Third, because PVC origin was categorized using ECG-derived patterns and invasive electroanatomic mapping was not available in most cases, some degree of localization uncertainty is unavoidable. Consistent with these considerations, the distribution of PVC origin did not differ significantly between patients with and without myocardial changes (Table 3).

### Pathogenesis of myocardial changes in patients with idiopathic PVCs

The finding of subclinical myocardial changes on CMRI in patients who otherwise appear normal on ECG, echocardiography raises the question: what is the nature and cause of these myocardial changes? Two broad possibilities can be considered. First, these patients might be in an early, subclinical stage of a primary cardiomyopathic process. Two patients in our study had CMRI findings (intramyocardial fat on T1-weighted images with high native T1) suspicious for arrhythmogenic cardiomyopathy. It is possible that with longer follow-up, some of these patients could develop more definitive signs of cardiomyopathy. Second, frequent PVCs themselves might induce structural changes in the myocardium over time. There is some evidence to support this “PVC-induced cardiomyopathy” concept: Chung et al. observed that in patients with hypertrophic cardiomyopathy, a higher PVC burden was associated with expansion of the extracellular space (higher ECV), suggesting PVCs may contribute to diffuse fibrosis (16). Similarly, Walters et al. demonstrated in a swine model that chronic ventricular ectopy led to greater ventricular fibrosis compared to sinus tachycardia at the same rate (17). In our study, PVC burden alone was not significantly linked to fibrosis, but the presence of NSVT and longer runs of PVCs were. This might imply a threshold effect or that more complex arrhythmias (not just frequent single PVCs) are needed to cause structural remodeling.

It remains to be determined whether the myocardial changes seen on CMRI in PVC patients are reversible. If frequent PVCs are the culprit, then successful elimination of PVCs might lead to improvement or normalization of T1/ECV values over time. Studies that perform CMRI before and after PVC ablation could shed light on this issue.

### Limitations

There were several limitations in the present study. First, since this was a retrospective study, selection bias in the indication for CMRI at the time of imaging cannot be completely excluded, which may have inflated the prevalence of myocardial changes. At our institution, for patients with frequent PVCs we generally perform CMRI in all cases—provided there are no contraindications—irrespective of whether ECG and echocardiography show abnormalities; thus, the degree of bias is likely limited. Nonetheless, a prospective study will be needed to validate these findings. Second, although we excluded cases with multifocal PVCs, prior studies have shown that multifocal PVCs are more likely to reflect underlying structural heart disease. In the present study, we excluded these patients in order to restrict our cohort to idiopathic PVCs that might otherwise be considered benign on routine follow-up. As an additional potential source of bias, the exclusion of patients with multifocal PVCs may have selectively removed individuals with occult structural heart disease, thereby underestimating the prevalence of myocardial changes on CMRI and limiting the generalizability of our findings (18). Third, during a median follow-up of 375 days (131–1,573 days), no cardiovascular events occurred in any of the patients, irrespective of myocardial changes on CMRI. This may reflect the relatively short follow-up and small sample size, which limit our ability to assess prognosis adequately. Therefore, these results do not allow us to conclude that outcomes are favorable despite myocardial changes on CMRI; larger, prospective studies with longer follow-up are needed. Fourth, the sample size was small, and PVC origins were heterogeneous and primarily determined using ECG-derived categories; because invasive electroanatomic mapping was not available in most cases, we were unable to perform meaningful origin-specific subgroup analyses or robust anatomical co-localization between PVC origin and CMRI abnormalities. Finally, we defined myocardial changes of T1 and ECV based on threshold values (1,270 ms and 30%, respectively) informed by literature and our scanner's normal reference values; however, optimal cut-offs for detecting pathology in this specific population are not well-established and might differ depending on technical factors. A larger prospective study would be valuable to confirm our results and to establish standardized CMRI criteria for early cardiomyopathy in the context of idiopathic PVCs.

## Conclusion

Even after excluding overt structural heart disease by ECG and echocardiography, CMRI—adding native T1 mapping, and ECV—frequently revealed subclinical myocardial changes in patients with idiopathic PVCs. Patients with myocardial changes on CMRI tended to be older and were more likely to exhibit NSVT and longer runs of PVCs. These results suggest that CMRI can unmask subclinical myocardial changes in idiopathic PVC patients and might help identify a subset of patients who warrant closer monitoring. Given the small sample size and heterogeneous, ECG-based estimation of PVC origin, these findings should be considered hypothesis-generating, and larger prospective studies with detailed electro-anatomical mapping and longer follow-up are needed.

## Data Availability

The raw data supporting the conclusions of this article will be made available by the authors, without undue reservation.
